# Home-Based Primary Care in the Department of Veterans Affairs: History, Lessons Learned, and Next Steps

**DOI:** 10.1093/geroni/igaf122.2441

**Published:** 2025-12-31

**Authors:** Brooke Jespersen, Anaïs Tuepker, Elizabeth Hulen, Senta Wiederholt, Dorothea Murray, Samuel Edwards

**Affiliations:** VA Portland Health Care System, Portland, Oregon, United States; VA Portland Health Care System, Portland, Oregon, United States; VA Portland Health Care System, Portland, Oregon, United States; VA Portland Health Care System, Portland, Oregon, United States; Oregon Health & Science University, Portland, Oregon, United States; VA Portland Health Care System, Portland, Oregon, United States

## Abstract

Launched in 1970, the Department of Veterans Affairs (VA) Home-Based Primary Care (HBPC) Program is a distinctive, well-resourced home care model that has broadly influenced the development of HBPC practice. VA HBPC delivers team-based, comprehensive, longitudinal primary care in the homes of Veterans who are unable to access or have great difficulty accessing clinic-based care. The program’s goal is to promote optimum health, while reducing the need for hospital, emergency room, and nursing home care. Overall, VA HBPC has produced favorable outcomes (e.g. reduced hospital, emergency, and nursing home utilization; high patient and caregiver satisfaction) and has lowered health care costs. Given population aging, there will be more medically complex older people who would benefit from home-based care; yet most do not have access to an HBPC program. It is critical that VA and non-VA health systems expand and improve HBPC to meet the high needs of this patient sector in a patient-centered, yet cost-effective manner. VA HBPC’s fifty-five-year history contains important lessons about the provision of home-based medical care for high risk, high needs patients. In this presentation, we draw on the literature and our institutional knowledge as VA HBPC researchers to provide a narrative history of VA HBPC. We highlight key innovations, identify challenges to program expansion and care delivery, and propose next steps for research and practice, with the goal of informing home-based medical care in VA and beyond.

